# The Association Between Reorganization of Bilateral M1 Topography and Function in Response to Early Intensive Hand Focused Upper Limb Rehabilitation Following Stroke Is Dependent on Ipsilesional Corticospinal Tract Integrity

**DOI:** 10.3389/fneur.2019.00258

**Published:** 2019-03-26

**Authors:** Mathew Yarossi, Jigna Patel, Qinyin Qiu, Supriya Massood, Gerard Fluet, Alma Merians, Sergei Adamovich, Eugene Tunik

**Affiliations:** ^1^Movement Neuroscience Laboratory, Department of Physical Therapy, Movement and Rehabilitation Science, Bouve College of Health Sciences, Northeastern University, Boston, MA, United States; ^2^SPIRAL Group, Department of Electrical and Computer Engineering, Northeastern University, Boston, MA, United States; ^3^Department of Rehabilitation and Movement Sciences, School of Health Professions, Rutgers Biomedical and Health Sciences, Newark, NJ, United States; ^4^Brookdale Rehabilitation – North Campus, Naples Community Hospital, Naples, FL, United States; ^5^Department of Biomedical Engineering, New Jersey Institute of Technology, Newark, NJ, United States; ^6^Department of Bioengineering, College of Engineering, Northeastern University, Boston, MA, United States; ^7^Department of Electrical and Computer Engineering, College of Engineering, Northeastern University, Boston, MA, United States

**Keywords:** stroke, upper limb, subacute, virtual reality, robotics, transcranial magnetic stimulation

## Abstract

Transcranial magnetic stimulation (TMS) induced motor evoked potentials (MEPs) are an established proxy of corticospinal excitability. As a binary measure, the presence (MEP+) or absence (MEP-) of ipsilesional hemisphere MEPs early following stroke is a robust indicator of long-term recovery, however this measure does not provide information about spatial cortical reorganization. MEPs have been systematically acquired over the sensorimotor cortex to “map” motor topography. In this investigation we compared the degree to which functional improvements resulting from early (<3 months post-stroke) intensive hand focused upper limb rehabilitation correlate with changes in motor topography between MEP+ and MEP- individuals. Following informed consent, 17 individuals (4 Female, 60.3 ± 9.4 years, 24.6 ± 24.01 days post first time stroke) received 8 one hour-sessions of training with virtual reality (VR)/Robotic simulations. Clinical tests [Box and Blocks Test (BBT), Wolf Motor Function Test (WMFT), Upper Extremity Fugl-Meyer (UEFMA)], kinematic and kinetic assessments [finger Active Range of Motion (finger AROM), Maximum Pinch Force (MPF)], and bilateral TMS mapping of 5 hand muscles were performed prior to (PRE), directly following (POST), and 1 month following (1M) training. Participants were divided into two groups (MEP+, MEP-) based on whether an MEP was present in the affected first dorsal interosseous (FDI) at any time point. MEP+ individuals improved significantly more than MEP- individuals from PRE to 1M on the WMFT, BBT, and finger AROM scores. Ipsilesional hemisphere FDI area increased significantly with time in the MEP+ group. FDI area of the contralesional hemisphere was not significantly different across time points or groups. In the MEP+ group, significant correlations were observed between PRE-1M changes in ipsilesional FDI area and WMFT, BBT, and finger AROM, and contralesional FDI area and UEFMA and MPF. In the MEP- group, no significant correlations were found between changes in contralesional FDI area and functional outcomes. We report preliminary evidence in a small sample that patterns of recovery and the association of recovery to bilateral changes in motor topography may depend on integrity of the ipsilesional cortical spinal tract as assessed by the presence of TMS evoked MEPs.

## Introduction

Stroke is a leading cause of adult long-term disability in the United States and the financial burden of related care is among the fastest-growing expenses for Medicare (1). Proportionally more stroke survivors are left with upper extremity impairment and disability than that of the lower extremity (2). At 6 months post-stroke, about 30–60% of affected individuals do not regain functional use and only 5–20% achieve full return of arm function (3, 4). Recovery of hand function is notably impervious to intervention in part due to the complexity of motor control required for dexterous function. At six months post-stroke ~65% of affected persons continue to have hand deficits that profoundly affect their ability to perform their usual activities and affect their independence (2, 5); and only 5% of those with initial severe paresis will have full recovery (6). Importantly, impaired hand function is often the most disabling deficit for many post lesion (7).

Numerous investigations have provided evidence indicating rehabilitation interventions must be initiated early after stroke to maximize recovery (8, 9). Although the optimal time period is not clear, the first month post-stroke is a crucial time for plasticity (8). Yet the vast majority of studies on emerging therapeutic interventions have focused on individuals in the chronic phase after stroke with limited work looking at interventions during acute and sub-acute phases (10–12). In fact, as reported in a 2013 review, only 6% of all stroke motor rehabilitation clinical trials have enrolled all patients within the first 30 days after a stroke (9). In light of recent evidence for the greater effectiveness of early rehabilitation, this staggering statistic highlights the need for investigation of intensive hand focused upper limb rehabilitation initiated early after stroke.

Perhaps most important are investigations comparing changes in impairments, function and neurophysiology early following stroke to identify the biomarkers of recovery. Transcranial Magnetic Stimulation (TMS) induced motor evoked potentials (MEPs) are an established proxy of corticospinal excitability (13). Numerous previous investigations have found that the presence (MEP+) or absence (MEP-) of MEPs early after stroke is a robust indicator of long-term recovery (14, 15). More recently, Stinear (16) suggested that people without MEPs (MEP-) at 2 weeks post-stroke have “limited or no predicted potential for upper extremity recovery” at 12 weeks after stroke.

Though numerous studies have indicated that the presence or absence of MEPs may be a strong predictor of recovery, change in the distribution of activation indicating reorganization of motor topography may provide additional insight into patterns of recovery. MEPs can be acquired over the sensorimotor cortex such that the two-dimensional position of the coil over the scalp can be used to generate a multivariate excitability map akin to those classically acquired with invasive stimulation, albeit with lower resolution. Use of TMS mapping to track ipsilesional motor reorganization over the first months to 1 year following stroke has generally indicated that increased excitable area in the ipsilesional hemisphere was associated with recovery of the impaired hand (17–19), though other studies found no change in ipsilesional excitable area over the same period (20, 21). Association of better outcomes with expansion of ipsilesional cortex activation is in line with numerous findings in human and animal models [see (22, 23) for a review]. Two investigations using TMS mapping during this early time period found increased excitable area in the contralesional hemisphere was associated with poorer outcomes (17, 24). This finding is in contrast with a number of studies which did not find changes in contralesional hemisphere excitable area or associations between changes in contralesional hemisphere topography and recovery of function in the subacute period (18–20). The association between contralesional topographic reorganization and functional recovery is complex, with numerous conflicting findings in both human and animal models, indicating beneficial or maladaptive influence on function [see (22, 23) for a review]. Although, these studies provide some indication of the general pattern of recovery; it is equally important to investigate the changes in functional-structural associations during focused intervention.

Interventional studies in the chronic phase post-stroke have used TMS based mapping of the ipsilesional hemisphere to quantify the spatial patterns of recovery of the corticospinal system in MEP+ patients (25, 26); all noting an increase in the peak MEP and area of MEPs representing the hand in the ipsilesional sensorimotor cortex. To date, there have been few studies that have investigated the association of functional outcomes and TMS measures of cortical topography with intensive upper limb intervention in the early stages following stroke (27–30). Findings from Ro et al. (30), Boake et al. (28) and Sawaki et al. (29) [in which patients were enrolled either in the first 14 days (28, 30), or at 3 to 9 months (29)] indicate that increased area of excitation in the ipsilesional hemisphere is associated with increased functional improvement in individuals receiving Constraint Induced Movement Therapy (CIMT) compared to controls receiving usual care. Contrary to this finding, Platz et al. (27) did not find any change in the number of active sites in their two treatment groups (Bobath and BASIS training), though reduction in map area was shown in the usual care group (27). Ludemann-Posdubecka and Nowak (31) offer a comprehensive review of observation and interventional studies assessing TMS mapping of cortical hand motor representation as a marker for recovery of function after stroke. Overall, most studies have compared changes in motor topography to a limited set of clinical measures of function or impairment and no study to date has compared contralesional changes between those individuals who do and do not have ipsilesional MEPs.

In this investigation, we examined the relationship between changes in function/motor recovery and cortical motor topography in a group of patients undergoing early (<3 months) and intensive hand focused upper limb rehabilitation using the NJIT-RAVR, an integrated VR/Robotic platform that was shown to be effective at reducing impairments in a chronic stroke population (32–34). With the exception of one small study from our group, no study has yet examined this relationship post VR/Robotic training (35). Data was collected in preparation for a now ongoing randomized controlled trial (RCT) to study the effects of timing and dosing of VR/Robotic intervention, and results for the intervention group are presented to show feasibility for use of TMS to measure neurophysiological correlates of recovery. Because specific hand therapy, by and large, is a small percentage of therapy received in the subacute period in US rehabilitation practice, selection of subjects from the intervention group only ensured that each individual did indeed receive therapy and that the dosage, in actual movement repetitions, was roughly equal among our sample. Specifically, we tested the degree to which clinical, kinematic, and kinetic measures of functional improvement correlated with changes in bilateral motor cortical topography (assessed by TMS mapping) in individuals with and without preserved ipsilesional corticospinal integrity (also assessed with TMS). We hypothesized that functional improvements would be greater in MEP+ individuals and that an increase in ipsilesional cortical territory would correlate to markers of functional improvement. In a secondary analysis we compared functional and topographical changes in the contralesional hemisphere between individuals who were positive for the presence of MEPs in the ipsilesional hemisphere (MEP+) and those who were not (MEP–). We predicted greater expansion of contralesional cortical territory in MEP- individuals, and that the degree of expansion would be associated with worse outcomes in this cohort of subjects. An important and novel tertiary exploratory analysis of MEP “converters,” individuals who were MEP- at baseline and later converted to MEP+, was also carried out to understand how reinstatement of MEPs is related to functional recovery.

## Materials and Methods

### Subjects

Subjects were recruited from the in-patient rehabilitation department of a suburban hospital. After initial screening by the department's physician, a physical therapist screened subjects based on the following criteria: *Inclusion:* (1) within 3 months post-stroke, (2) between the ages of 30 and 80, (3) for the severely impaired group: categorized as Stage 1 on the Hand Impairment Inventory of the Chedoke-McMaster Stroke Assessment (36), for the moderately impaired group: have partial active shoulder flexion, or abduction, elbow extension and wrist extension against gravity, and trace extension at the fingers (detected visually) that can be reproduced several times in a minute. *Exclusion:* (1) severe spasticity [Modified Ashworth score of 3 or higher (37)], (2) cognitive deficits rendering them unable to follow three step commands or attend to a task for at least 10 min (3) hemispatial neglect rendering them unable to interact with an entire 24 inch computer monitor—positioned at midline, (4) proprioceptive loss that rendered a potential subject unable to interact with a virtual environment without looking at their hand (5) unstable blood pressure and oxygen saturation responses to activity. A separate screening and consent process for the motor mapping evaluation using TMS was conducted. Exclusion criteria for TMS included metallic or electronic implants in the head, pregnancy, and history of epilepsy.

### Training Protocol and Schedule of Outcomes Assessment

All subjects participated in 8 sessions over a 2-week period. In each session, subjects trained for 1 h using the NJIT-RAVR system interfaced with virtual reality simulations. Additionally, all subjects participated in their on-going in/out-patient physical, occupational and speech therapy. Clinical, kinematic/kinetic, and TMS evaluations were performed on the day prior to beginning training (PRE), the day following the last day of training (POST), and 1 month thereafter (1M).

### Description of the VR/Robotic System

**Figure 1:**
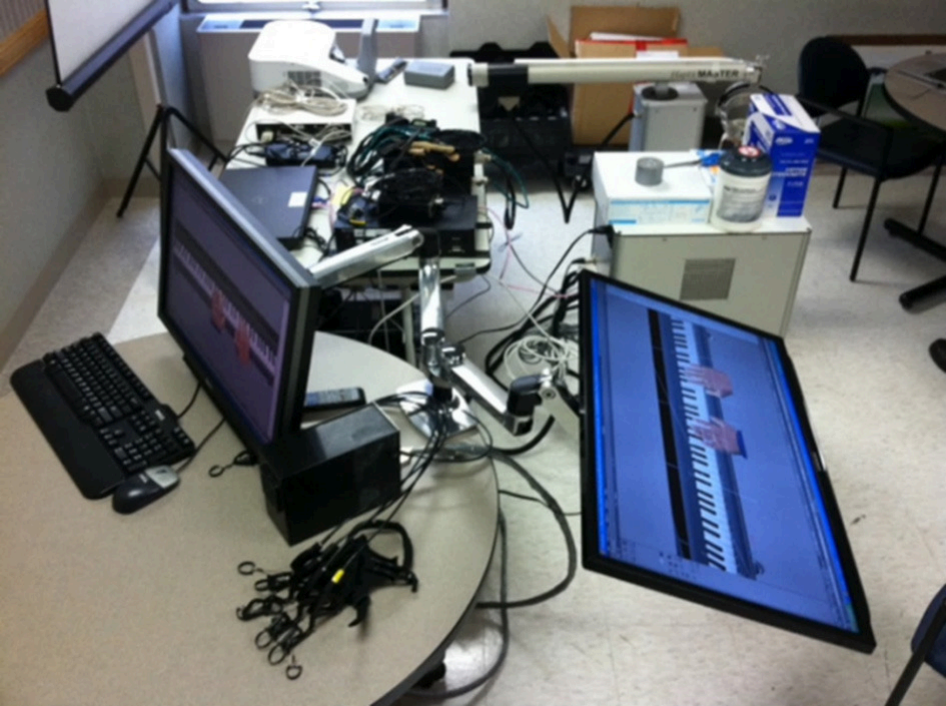
NJIT-RAVR system.

The NJIT-RAVR system is comprised of an arm training robot (Haptic Master [Moog NCS, The Netherlands]) combined with a 3 degree of freedom gimbal, and an integrated system for the hand that consists of an instrumented measurement glove (CyberGlove [Immersion, USA]), a cable actuated hand exoskeleton that facilitates finger extension for those persons with more severe impairment (CyberGrasp [Immersion, USA], and a 3-dimensional magnetic tracking system that tracks hand and arm position (TrackSTAR™ [(Ascension Technology, USA])—the NJIT Track–Glove System. The system utilizes an ATI Nano17™ force sensor (ATI Industrial Automation, USA) for pinch force measurement. The Haptic Master was individually programmed to provide assistance to lower functioning subjects with progressive adaptations that lessened the help provided as subjects improved over time. Please refer to Adamovich et al. (38) and Fluet et al. (39) for detailed information on this system (Figure 1).

### Description of Simulations and Targeted Hand Training

The VR environment was developed with Virtools 4.0 software package (Dassault Systemes, France) and a VRPack Plug-in that communicates with an open source Virtual Reality Peripheral Network (VRPN) interface. The NJIT-RAVR robotic system that interfaces with our suite of impairment and activity based virtual reality simulations was used to train the hand and arm separately. Individuals with moderate initial impairment were provided training comprising three hand and three proximal arm simulations ~10 min on each of the six simulations during each session. The hand simulations consisted of the Virtual Piano, Monkey Business, and Space Pong games, and the arm simulations were the Cups, Hammer, and Space Ship games (32, 33). Individuals with severe deficits were provided with a modified training protocol consisting of two types of priming (virtual mirror feedback and contralaterally controlled hand opening) to prime the motor cortex and reinforce motor networks in the lesioned hemisphere (40) prior to training their affected hand with a force modulation task (41).

### Clinical Outcome Measures

Three clinical tests measuring functional and impairment based deficits were performed by a physical therapist: (1) Wolf Motor Function Test (WMFT), a time-based series of tasks to evaluate upper extremity function (42), (2) the upper extremity portion of the Fugl-Meyer Assessment of Sensorimotor Recovery After Stroke (UEFMA), a task performance exam that assesses motor impairment after stroke (43, 44), and (3) the Blocks and Box test (BBT), a unilateral assessment of gross manual dexterity (45).

### Kinematic and Kinetic Measures

Finger angles were collected using a CyberGlove™ (Immersion, USA). Finger range of motion (finger AROM) was measured as the difference between all of the joint angles with the fingers in a maximal actively flexed position and the joint angles of all of the fingers in a maximal actively extended position. Larger differences indicated better active finger range of motion. Pinch force was measured with an ATI Nano17™ force sensor (ATI Industrial Automation, USA) as the maximum voluntary pinch force (MPF) a subject can exert on a force sensor held between their paretic thumb and index finger, given two trials. Higher numbers indicate stronger pinch grip.

### TMS Mapping Procedure

Subjects were seated with their arm, hand, and fingers comfortably secured in a brace to limit motion. Surface electromyographic activity (EMG, Delsys Trigno, 2 kHz) was recorded from 5 muscles of the limb contralateral to stimulation side (first dorsal interosseus [FDI], abductor pollis brevis [APB], abductor minimi [ADM], flexor digitorum superficialis [FDS], and the extensor digitorum communis [EDC]. To assure spatial TMS precision, each subject's head was coregistered to a canonical high-resolution anatomical MRI for frameless neuronavigation (Brainsight–Rogue Research, Canada). All TMS measures were taken at rest and background EMG was monitored to ensure that muscles remained relaxed. The TMS coil (Magstim, 70 mm double coil) was held tangential to the scalp, with the handle posterior 45° off the sagittal plane (46). Motor evoked potentials (MEPs) were sampled until the loci with the largest MEP was located (14, 47). This method has been shown to have high intra and inter experimenter reliability (47), has been cross-validated with fMRI, and is robust in identifying the loci of greatest activation for a given muscle (48). Resting motor threshold (RMT) was determined at this location as the minimum intensity required to elicit MEPs >50 uV in the FDI muscle on 50% of 6 consecutive trials (49). The choice of intensity, 110% FDI RMT, represented a compromise between the different excitability thresholds of the selected muscles, as has been done previously in other studies investigating multi-muscle topography using TMS (17, 50). The hotspot and threshold were determined at each mapping session. All mapping was performed with the subject at rest and stimulation intensity set to 110% of the determined RMT (51). A 7 × 7 cm area surrounding the motor hotspot was marked using the neuronavigation software to provide consistent map boundaries. TMS pulses (150) were delivered at a 4 s interstimulus interval within the bounds with special attention paid to regions surrounding the hotspot territory. Real time feedback of multi-muscle MEPs and neuronavigated coil position was used to maximize the map information obtained by increasing the density of points in excitable and border regions while giving less attention in far-away non-responsive areas (52). Mapping procedures were conducted for both the ipsilesional and contralesional hemispheres (Figure 3). For each stimulation point the motor evoked potential (MEP) was calculated as the peak-to-peak amplitude of the EMG signal 20–50 ms after the TMS pulse.

### TMS Mapping Analysis

A threshold of 50 uV was used to identify MEPs from background EMG (51). To allow comparisons across maps and sessions, MEP amplitudes and stimulation points were interpolated to a 7 × 7 cm mesh of 0.375 mm resolution, centered around the M1 hotspot, using cubic surface interpolation (54, 55). Extent of the representation producing corticospinal output (MEPs) for individual muscle, or map area, was calculated using double trapezoidal integration of the interpolated map (35). Map area has been used extensively to describe sensorimotor cortex reorganization after stroke [for a review see Cortes et al. (56)]. Furthermore, a recent systematic review of the use of TMS as an outcome measure for rehabilitative interventions found that map area was the most likely measure to correlate to changes in clinical outcomes (57).

### Statistical Analysis

Ipsilesional hemisphere maps were analyzed with a one-way repeated measures ANOVA, with a within factor of Time (PRE, POST, 1M). Significant findings were further analyzed using *post-hoc* paired comparisons with Bonferroni correction for multiple comparisons. Contralesional hemisphere maps, resting motor threshold, kinematic, and clinical outcomes were each analyzed with a two-way mixed factorial ANOVA, with a within factor of Time (PRE, POST, 1M) and a between factor of ipsilesional MEP presence (MEP+, MEP–). Significant interactions were analyzed with independent samples *t*-test to test for differences at PRE, and to test for differences in PRE-POST, POST-1M, and PRE-1M change scores. To test the relationship between M1 changes and function, PRE-1M changes in FDI area were correlated to PRE-1M changes in clinical, kinematic, and kinetic measurements. Alpha level was set at 0.05.

## Results

Seventeen individuals (4 Female, 60.3 ± 9.4 years, 24.64 ± 24.01 days post CVA) with first time stroke participated in the intervention. Participant characteristics are listed in Table 1. All training was well-tolerated without adverse events or fatigue that had a negative impact on their rehabilitative program in or out of the intervention.

**Table 1:** Participant characteristics.

**Subject**	**Group**	**Age**	**Sex**	**Days post stroke**	UEFMA/Severity^*^ At PRE	**Lesion location**
S1	MEP+	62	F	39	25/Mod	R parietal
S2	MEP+	62	M	92	27/Mod	R MCA
S3	MEP+	45	M	12	32/Mod	L Putamen
S4	MEP+	62	F	6	47/Mod	L MCA
S5	MEP+	76	M	7	33/Mod	R frontal, parietal, temporal
S6	MEP+	70	M	10	37/Mod	R MCA
S7	MEP+	60	M	7	11/Severe	R periventricular white matter
S8	MEP+	53	M	13	21/Mod	L temporal, parietal
S9	MEP+	65	F	5	3/Severe	R pons
Mean (SD)		61.7 (9.0)		21.2 (28.5)	26.2 (14.3)	
S10	MEP–	76	M	54	46/Mod	L pons
S11	MEP–	63	M	68	41/Mod	R pons
S12	MEP–	64	M	29	44/Mod	R - unknown
S13	MEP–	43	F	7	31/Mod	L MCA/ACA
S14	MEP–	66	M	10	3/Severe	R basal ganglia
S15	MEP–	53	M	7	4/Severe	L MCA
S16	MEP–	55	M	9	2/Severe	R PLIC
S17	MEP–	51	M	44	6/Severe	R basal ganglia
Mean (SD)		58.9 (10.4)		28.50 (24.2)	22.1 (20.2)	

* *Based on Woodbury et al. (53). Woodbury classification was calculated post-hoc and was not used for stratification into moderate and severe groups*.

Participants were divided into two groups (MEP+, MEP–) based on whether TMS to the ipsilesional hemisphere produced an MEP in the affected FDI muscle at rest. Participants stratified into the MEP+ group included all individuals for whom a response could be elicited from the FDI at any time point (PRE, POST or 1M). Of these individuals 5/9 participants were MEP+ at baseline, and 4/9 “converted” to MEP+ at either POST or 1M. Participants in the MEP- group were those individuals for whom a response could not be elicited from the FDI at all time points (PRE, POST, and 1M). Analysis was performed for the ipsilesional hemisphere in the MEP+ group only (the MEP- group did not have ipsilesional MEP responses to analyze), and the contralesional hemisphere in both MEP+ and MEP− groups. There was no statistical difference in the days post-stroke between MEP+ (21.22 ± 28.51) and MEP– (28.5 ± 24.19) participants [*t*_(15)_ = −0.56, *p* = 0.582]. There was also no statistical difference in the baseline UEFMA scores between MEP+ (26.22 ± 14.3) and MEP– (22.13 ± 20.2) participants [*t*_(15)_ = 0.50, *p* = 0.62] (Table 1).

### Corticospinal Integrity, Impairment, and Function

Mixed factorial ANOVAs with factors Time (PRE, POST, 1M) and Group (MEP+, MEP–) were used to test for main effects and interactions in clinical, kinematic, and kinetic outcomes. A main effect of Time was significant for all measured outcomes indicating that impairment level decreased over time. Group main effect was significant for finger AROM only [*F*_(1, 15)_ = 9.94, *p* = 0.028]. Time X Group interactions were significant for the WMFT, BBT, and finger AROM evaluations. Significant interactions were followed with independent samples comparisons between groups to test for differences at PRE and differences in the amount of change from PRE to POST, and PRE to 1M. *Post-hoc* independent comparisons revealed no significant differences between groups at PRE for any outcome, indicating baseline function was similar between groups. There were significant between Group differences in the amount of change from PRE to POST in the WMFT [*t*_(11.02)_ = −2.22, *p* = 0.048], BBT [*t*_(12.12)_ = 2.25, *p* = 0.044], and finger AROM [*t*_(11.66)_ = 2.29, *p* = 0.04], and from PRE and 1M in the WMFT [*t*_(15)_ = −3.44, *p* = 0.004], BBT [*t*_(15)_ = 2.66, *p* = 0.018], and finger AROM [*t*_(10.13)_ = 3.19, *p* = 0.01] indicating that MEP+ participants improved more than the MEP- participants for both time periods (Tables 2a,b).

**Table 2a:** Mixed factorial ANOVA outcomes for MEP+ and MEP- groups compared across time on clinical, kinematic, and kinetic measures.

**Test**	**Time**	**Group**	**TIME X Group**
Log WMFT	*F*_(2, 30)_ = 28.98 *p* < 0.001	*F*_(1, 15)_ = 0.18 *p* = 0.676	*F*_(2, 30)_ = 7.73 *p* = 0.002
BBT	*F*_(1.30, 19.47)_ = 23.94 *p* < 0.001^*^	*F*_(1, 15)_ = 1.72 *p* = 0.210	*F*_(1.30, 19.47)_ = 6.14 *p* = 0.017^*^
UEFMA	*F*_(2, 30)_ = 51.42 *p* < 0.001	*F*_(1, 15)_ = 1.42 *p* = 0.252	*F*_(2, 30)_ = 2.06 *p* = 0.146
Finger AROM	*F*_(2, 30)_ = 7.26 *p* = 0.003	*F*_(1, 15)_ = 9.94 *p* = 0.028	*F*_(2, 30)_ = 5.12 *p* = 0.012
Max Pinch Force	*F*_(2, 30)_ = 14.14 *p* < 0.001	*F*_(1, 15)_ = 0.15 *p* = 0.701	*F*_(2, 30)_ = 0.19 *p* = 0.822

* *Greenhouse Geisser corrected*.

**Table 2b:** *Post hoc* outcomes on clinical, kinematic, and kinetic measures for PRE – POST and PRE – 1M change scores between groups.

**Test**	**Pre – post**	**Pre – 1 month**
Log WMFT	*t*_(11.02)_ = −2.22 *p* = 0.048^*^	*t*_(15)_ = −3.44 *p* = 0.004
BBT	*t*_(12.12)_ = 2.25 *p* = 0.044^*^	*t*_(15)_ = 2.66 *p* = 0.018
UEFMA	*t*_(15)_ = 1.67 *p* = 0.116	*t*_(15)_ = 1.76 *p* = 0.099
Finger AROM	*t*_(11.66)_ = 2.29 *p* = 0.04^*^	*t*_(10.13)_ = 3.19 *p* = 0.01^*^
Max Pinch Force	*t*_(15)_ = 0.02 *p* = 0.981	*t*_(15)_ = 0.56 *p* = 0.586

* *Levene's test for equality of variances significant*.

The MEP+ group included both individuals who were MEP+ at baseline (*n* = 5) and individuals who became MEP+ at a later time point (*n* = 4). To date, few studies have addressed these “converters” and no study has specifically compared recovery between those who are MEP+ at baseline and those who convert to MEP+ at a later time. We performed a subanalysis looking at the differences in clinical and functional recovery measures between those who were MEP+ at baseline and those who were baseline-negative but converted to MEP+ over time. Mixed ANOVAs found no statistical differences or interaction effects between these two groups over time and provided justification for combining the two subgroups into one cohort: WMFT: [*F*_(2, 14)_ = 0.949, *p* = 0.411]; Finger AROM: [*F*_(1.202, 8.417)_ = 0.083, *p* = 0.824]; BBT: [*F*_(1.22, 8.51)_ = 2.77, *p* = 0.13]; UEFMA: [*F*_(2, 14)_ = 1.8942, *p* = 0.195]; MPF: [*F*_(2, 14)_ = 1.055; 0.374]. However, comparisons after stratification into subgroups should be interpreted with caution due to limited sample size.

### Ipsilesional and Contralesional Resting Motor Threshold

**Figure 2:**
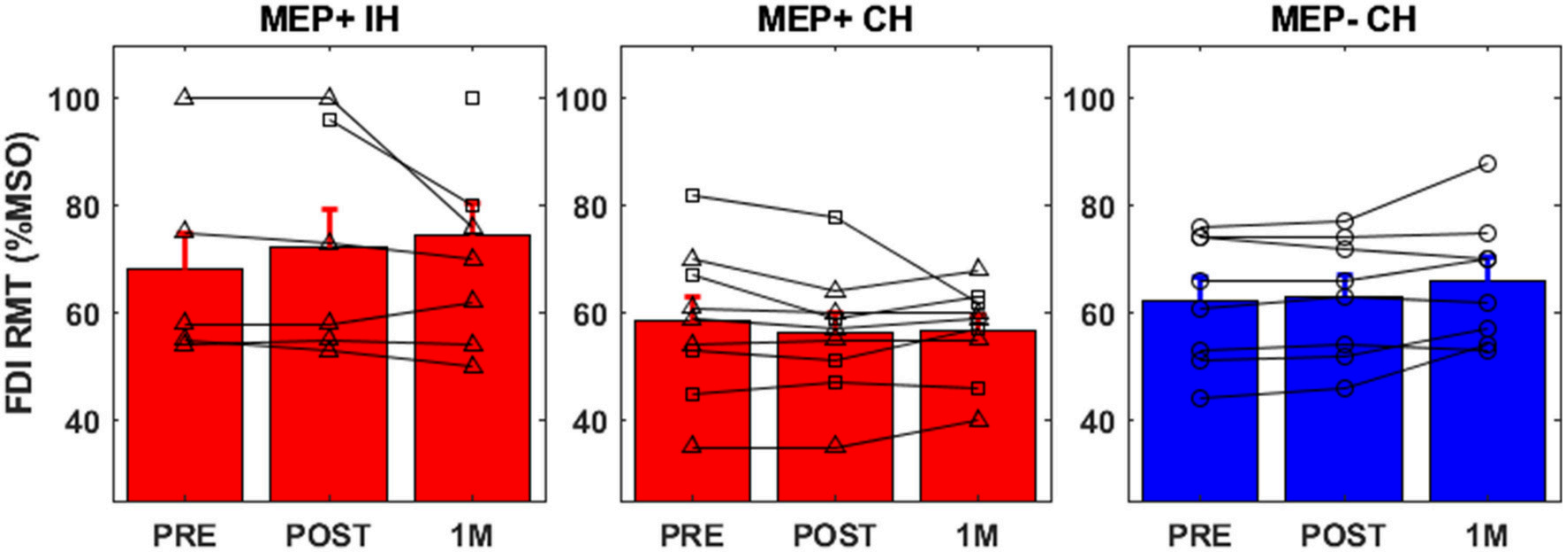
FDI resting motor threshold for all groups [Ipsilesional Hemisphere (IH), Contralesional Hemisphere (CH)]. Individuals who were MEP+ at baseline are denoted with triangle markers, individuals who converted to MEP+ at POST or 1M are denoted with square markers, and MEP- individuals are denoted by circular markers.

Individual resting motor thresholds for the ipsilesional (MEP+ only) and contralesional hemispheres at each time point are reported in Figure 2. Contralesional resting motor thresholds were generally consistent across sessions and accordingly a 2 × 3 ANOVA with factors of Group (MEP+/MEP-) and TIME (PRE, POST, 1M) produced no significant main effects or interactions. Ipsilesional resting motor thresholds of MEP+ individuals were higher than those found in the contralesional hemisphere for 7/9 subjects including all subjects that converted to MEP+ status at either POST or 1M. At 1M, when data were available for all participants, an independent samples *t*-test confirmed significantly higher iplsilesional than contralesional resting motor threshold [*t*_(16)_ = −2.714, *p* = 0.015]. Also at 1M, there was a significant difference in resting motor threshold between those individuals who we MEP+ at PRE and those individuals who converted to MEP+ at a later time [*t*_(7)_ = 3.697, *p* = 0.008]. Absence of MEPs at 100% of stimulator output prevented the correlation of ipsilesional motor threshold to measures of impairment and function. There were no statistically significant correlations between contralesional resting motor threshold and any measure of impairment or function in either MEP+ or MEP- individuals.

**Figure 3:**
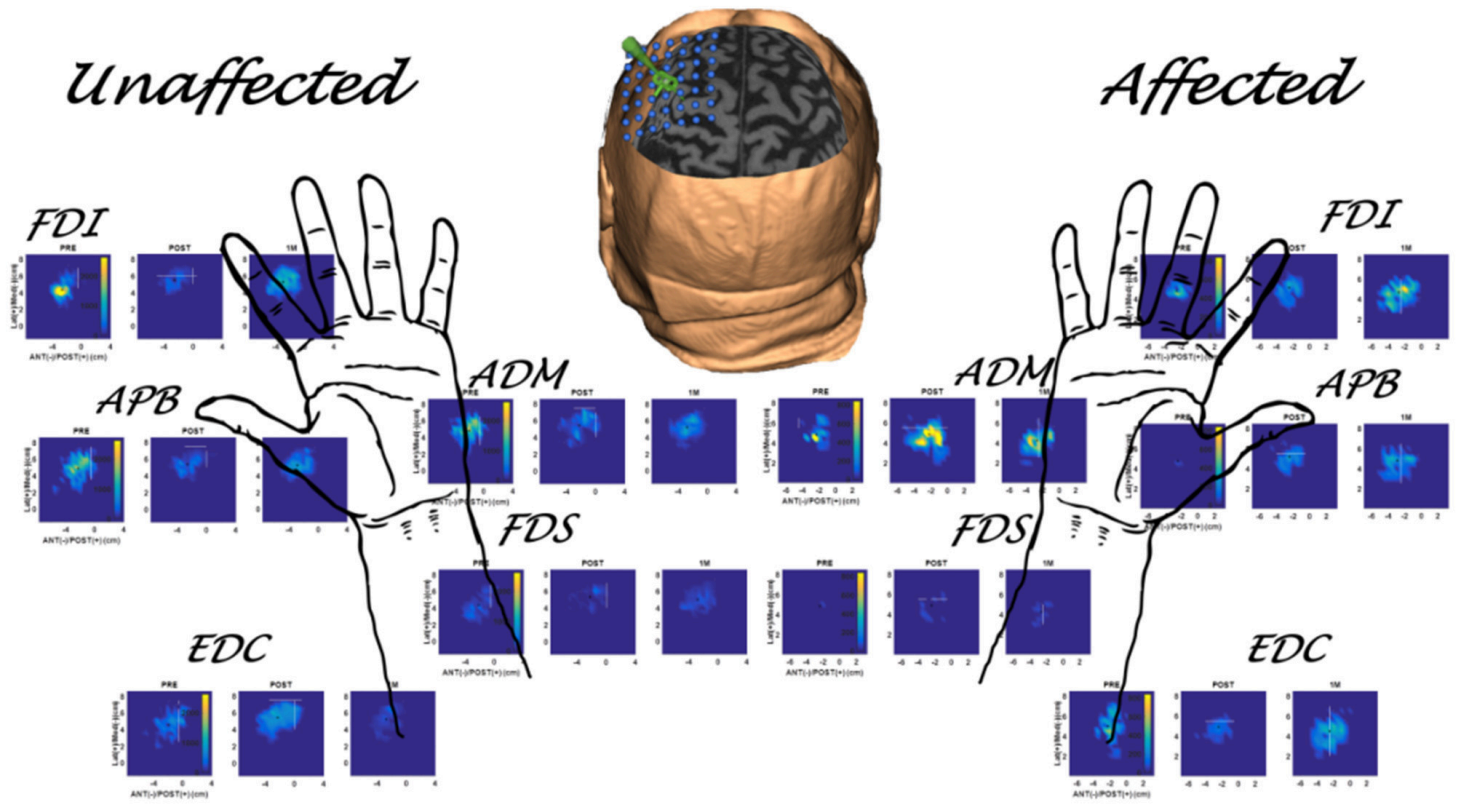
Multiple muscle mapping data for the unaffected hand (contralesional hemisphere) and affected hand (ipsilesional hemisphere) of a representative subject (S1 in Table 1) in the MEP+ group. PRE, POST, and 1M maps are presented for each muscle.

### Ipsilesional and Contralesional Motor Topography

**Figure 4:**
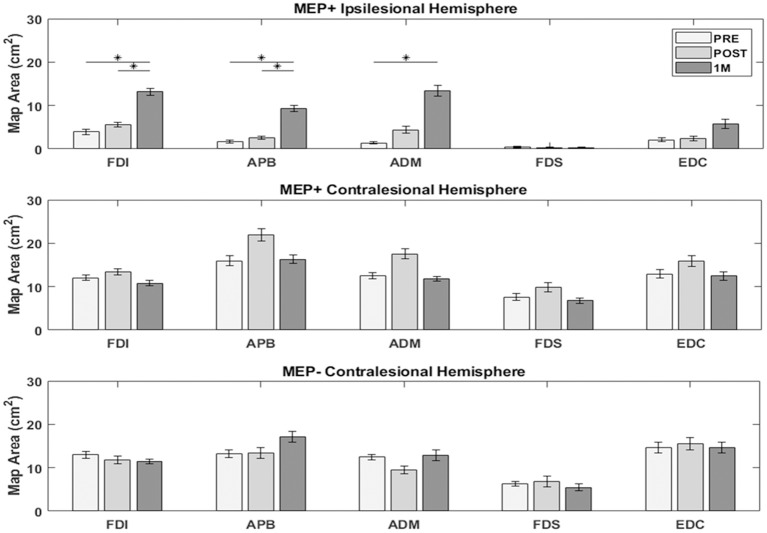
Excitable cortical area for each muscle. Intrinsic muscle map area in the ipsilesional hemisphere of MEP+ participants increased significantly over the measured time period. Changes were less notable, or absent in the contralesional hemisphere for both groups. (^*^) indicates significant differences between time points (*p* < 0.05).

In the ipsilesional hemisphere of the MEP+ group, excitable territory for upper limb muscles increased steadily in the period from PRE to 1M, with greater changes appearing in the intrinsic (FDI, APB, ADM) than extrinsic finger muscles [FDI (7.67 ± 10.4), APB (6.61 ± 5.6), ADM (10.9 ± 12.4), FDS (0.11 ± 1.1), EDC (4.67 ± 8.9)]. In the contralesional hemisphere of the MEP+ group, the excitable territory for all five muscles showed an increase from PRE to POST and decrease from POST to 1M (non-significant change at both time frames). Changes in the contralesional hemisphere of the MEP- group were more variable and were characterized by minimal change across measurement times at the group level (Figure 4).

Repeated measures ANOVAs testing ipsilesional hemisphere map area changes for each muscle of the MEP+ group revealed a significant effect of Time for the FDI [*F*_(2, 16)_ = 7.84, *p* = 0.004], APB [*F*_(2, 16)_ = 12.57, *p* = 0.001], and ADM [*F*_(2, 16)_ = 6.41, *p* = 0.009]. Pairwise *post-hoc* comparisons indicated a significant increase in map area between PRE and 1M for the FDI [*t*_(8)_ = −3.37, *p* = 0.016], APB [*t*_(8)_ = −3.63, *p* = 0.007], and ADM [*t*_(8)_ = −3.05, *p* = 0.016], and between POST and 1M for the FDI [*t*_(8)_ = −2.37, *p* = 0.022] and APB [*t*_(8)_ = −3.81, *p* = 0.005]. EDC and FDS map area changes were not significant. Mixed factorial ANOVAs to test for changes in map areas in the contralesional hemisphere with factors Time (PRE, POST, 1M) and Group (MEP+, MEP–) indicated no significant main effects of Time or Group and no Time X Group interaction for all muscles tested.

**Figure 5:**
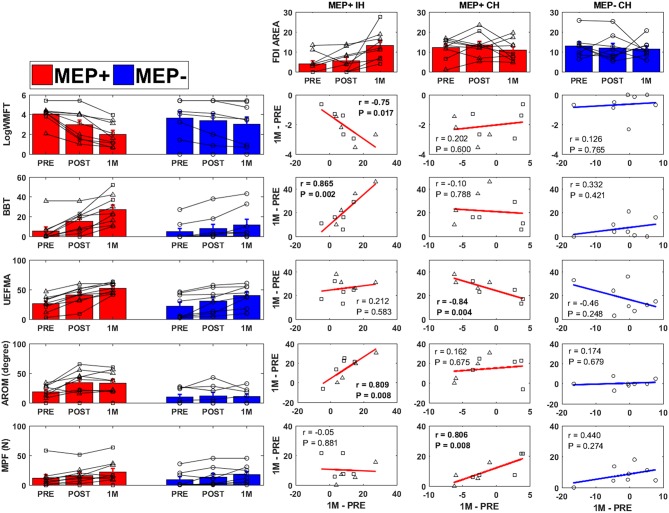
Clinical, kinematic, and FDI area changes for all groups [Ipsilesional Hemisphere (IH), Contralesional Hemisphere (CH)]. Individuals who were MEP+ at baseline are denoted with triangle markers, individuals who converted to MEP+ at POST or 1M are denoted with square markers, and MEP− individuals are denoted by circular markers. PRE to 1 Month change in FDI area (top row) was correlated to the change in WMFT (2nd row), BBT (3rd row), UEFMA (4th row), finger AROM (5th row), and MPF (6th row). Significant correlations are indicated in bold.

PRE to 1M changes in FDI area were correlated to changes in clinical, kinematic, and kinetic outcomes over the same period (Figure 5). In the ipsilesional hemisphere of the MEP+ group, significant correlations were observed between changes in FDI area and changes in the WMFT (*r* = −0.75, *p* = 0.017), BBT score (*r* = 0.865, *p* = 0. 002), and finger AROM (*r* = 0.809, *p* = 0.008. Contralesional hemisphere FDI area change in the MEP+ group was significantly correlated to the change in UEFMA (*r* = −0.84, *p* = 0.004) and MPF (*r* = 0.806, *p* = 0.008). No significant correlations were found between contralesional hemisphere cortical changes and clinical, kinetic, or kinematic outcomes for the MEP- group.

## Discussion

Recovery of neural function post-stroke is a complex process that involves initial reversal of diaschisis and activation of cell repair followed by changes in axonal sprouting in existing neuronal pathways, and synaptogenesis with concomitant modification in the cortical excitability and somatotopic remapping (58). Critically, these recovery processes involve both hemispheres and are heightened in the first 3 months after stroke (59). In this study, 2 weeks of intensive VR/Robotic based hand focused/upper limb therapy was initiated in the first 3 months post-stroke with the aim of capitalizing on the natural recovery processes. TMS mapping was used to evaluate macro-level changes in ipsi- and contralesional reorganization of M1 topography for five hand muscles. FDI muscle map changes were compared to clinical, kinematic, and kinetic outcomes to determine any correlation between TMS map changes and upper limb motor recovery. In light of recent evidence that the presence or absence of MEPs in the ipsilesional hemisphere measured shortly after stroke is an important neurophysiological biomarker of recovery and outcomes (60), patterns of motor recovery in the paretic upper limb and contralesional TMS map changes were compared between individuals who had MEPs (MEP+) and those who did not have MEPs (MEP-) in the ipsilesional hemisphere.

### Corticospinal Integrity, Impairment, and Function

Clinical and functional measures were not significantly different between MEP+ and MEP- groups at baseline, and as in Stinear et al. (61) there was a wide range of improvement for any given baseline measure of an individual patient (61). Individuals who were MEP+ showed significantly greater improvement on the WMFT, BBT, and in finger AROM from PRE−1M compared to MEP- individuals. This finding is in agreement with recent investigations from Stinear and Byblow that show individuals who have MEPs early after stroke experience proportional recovery of ~70% of impairment, whereas individuals who are MEP- do not have a stereotypical pattern of recovery (62–64). Interestingly, differences were not significant for the UEFMA and maximum pinch force despite a general pattern of improvement similar to the other three measures.

Understanding the recovery patterns of individuals who are MEP- at baseline but convert to MEP+ at a later time point is a poignant topic and a key aim of our current RCT (https://clinicaltrials.gov, NCT03569059). Several studies (17, 18, 24) have included these individuals but did not report specific analyses or descriptions of converters. Instead, these individuals were grouped as MEP- for comparison of associations of contralesional hemisphere map changes and functional recovery between MEP+ and MEP- individuals. In those studies that provide at least some description of this cohort, conversion to MEP+ at a later time point has not always been found to indicate more favorable clinical improvement (21, 65, 66). However, conflicting reports exist and in two studies, individuals who gained MEP+ status at a later time point showed consistent clinical improvement (19, 20). In the data presented here, lack of significant differences between individuals who were MEP+ at baseline and those who became MEP+ at POST or 1M appear to be in agreement with these studies. Furthermore, the relationship between cortical expansion and changes in clinical and functional measures of recovery in the period of PRE-1M suggests these individuals may share more in common with individuals who are MEP+ at baseline (see Figure 5).

### Resting Motor Threshold

Resting motor threshold reflects efficiency of TMS to excite corticospinal neurons which is dependent on the excitability of individual neurons and their local density (67). Consistency of contralesional resting motor threshold across time and higher resting motor threshold in the ipsilesional hemisphere, in comparison to the contralesional hemisphere, are in line with previous investigations in stroke. High reliability of the resting motor threshold as well as ease of collection has made it the most-used TMS outcome measures in intervention studies post-stroke, however, a recent review found that the post-intervention change in resting motor threshold was significant in only 2 of the 11 investigations reviewed (57). We did not find significant differences in contralesional resting motor thresholds between individuals who were MEP+ and those who were MEP- as might be expected given previous evidence of reduced interhemispheric inhibition from the ipsilesional to contralesional hemisphere and/or greater contralesional compensations for lost function of ipsilesional hemisphere with greater ipsilesional damage (68). This may be due to the small sample size in the current study. Alternatively, it could be that the VR/Robotic intervention prevented contralesional compensation; however, without a usual care control group this interpretation remains speculative. Ipsilesional resting motor threshold among individuals who recovered MEPs at time points after PRE were higher than those recorded in individuals who were MEP+ at the initial assessment. This finding is in agreement with the findings of Delvaux et al. (21), and suggests that these individuals may have different recovery patterns from those individuals who are consistently MEP- and individuals who are MEP+ soon after stroke with resting motor threshold values in the range of the contralesional hemisphere.

### Reorganization of Motor Topography

Motor map area has been suggested to reflect a combination of corticospinal excitability and somatotopy of the targeted muscles' M1 representation (69). In absence of consistent large changes in resting motor thresholds, changes in map area were more likely associated with changes in motor somatotopy (29).

The ipsilesional hemisphere in the MEP+ group was marked by significant expansion of map area across testing sessions for the intrinsic hand muscles (FDI, APB, ADM) only. The VR/Robotic intervention that we used incorporated similar doses of training for both fine motor and gross motor tasks of the hand-arm, and therefore, it is unlikely that differences in map area changes between intrinsic and extrinsic hand muscles are due to a training-specific effect. Patterns of reorganization may inherently differ between the intrinsic hand muscles–which are known to have larger and more excitable representations when compared to the extrinsic hand muscles in healthy individuals. However, this result should be interpreted with caution as differences may have resulted from choosing the stimulation intensity for mapping based on the FDI RMT, which is likely lower than that of the EDC or FDS. Unfortunately, it would not have been feasible to collect multiple maps, each one relative to each muscle's activation threshold, so the decision was made to base the mapping on the most commonly reported muscle, the FDI.

Expansion of FDI and APB territories in the ipsilesional hemisphere of the MEP+ group were significant between the POST and 1M time points, but not between the PRE and POST time points. This result is surprising given evidence that spontaneous recovery related cortical plasticity decreases after the first month following stroke (8). It is possible that the 2 weeks intervention using virtual reality and robotics was able to change the pattern of neurophysiological recovery *post intervention*; however without a control comparison this remains speculative at this time. Our ongoing RCT, once completed, is designed to address this possibility.

The PRE-1M changes in ipsilesional FDI area were significantly correlated with clinical markers of recovery (WMFT, BBT, and finger AROM) over the same period (Figure 5). Current evidence indicates that unilateral ipsilesional M1 excitation is important for the recovery of dexterous movement post stroke [see (70) and references within]. Scores on the WMFT, BBT, and finger AROM are likely to improve with more coordinated control of the index finger, which is representative of a motor task requiring use of corticospinal projections from the contralateral primary motor cortex (71). Strong correlation of these measures to map expansion may indicate that map expansion is a marker of intracortical reorganization of muscle representations which has been shown to correlate to recovery in animal models (72, 73).

In contrast to the ipsilesional hemisphere of the MEP+ group, area changes in the contralesional hemisphere of the same group were smaller and more variable. A pattern of increased excitable area from PRE-POST followed by a return to PRE levels at 1M, as was reported by Chieffo et al. (17), was observed but not significant. Increased contralesional area was significantly correlated with poorer performance on the UEFMA, a finding that appears to correspond with the findings of Chieffo et al. (17). In contrast, increased pinch force was associated with greater expansion of the contralesional area. Evidence for bilateral activation in the production of high forces with one hand is well-established in studies of healthy individuals (74). It is possible that individuals who showed significant increases in force production were better able to access bilateral networks, but this may have had a negative effect on control of movement and therefore the inverse relationship between clinical tests of impairment (UEFMA) and force production.

Changes in cortical topography in the contralesional hemisphere of MEP- individuals were nominal and did not correlate to any of the five measures of motor recovery. This provides preliminary evidence that the functional recovery processes related to contralesional hemisphere reorganization in individuals without MEPs may be fundamentally different than in individuals who show intact ipsilesional corticospinal integrity. It is possible that intact signaling from the ipsilesional hemisphere may be necessary to reorganize contralesional pathways, and when signaling from the ipsilesional hemisphere is absent, these individuals become more reliant on subcortical pathways for movement at the cost of fine motor control (75). Further research is necessary to understand the complex role of the contralesional hemisphere in recovery of hand function following stroke.

## Conclusions

Individuals engaged in VR/Robotic based training in the acute to early subacute period (<3 months) (76) following stroke showed significant recovery of upper limb function. We report preliminary evidence in a small sample that patterns of recovery and the association of recovery to bilateral changes in motor topography may depend on integrity of the ipsilesional cortical spinal tract as assessed by the presence of TMS evoked MEPs. Functional recovery was greater in individuals who were MEP+, and was significantly correlated to ipsilesional and contralesional changes in excitable cortical territory for an intrinsic hand muscle. Specific correlations were indicative that ipsilesional map expansion may be associated with increased manual dexterity, while contralesional change may be associated with strength. A subanalysis comparing those who were MEP+ at PRE and those who “converted” to MEP+ at POST or 1M found no differences in clinical or functional outcomes between the two groups. However, higher resting motor threshold at 1M in converters may indicate some fundamental difference from early MEP+ individuals. Individuals who were MEP- showed smaller and more variable patterns of recovery and no correlation between function outcomes and changes in contralesional map topography indicating the possible use of non-cortical compensatory pathways. Findings of the study were limited by small sample size and lack of a comparative control group. Given these limitations, interpretation was limited to the association between map changes and clinical and functional outcomes, and the prognostic value of early post-stroke mapping was not discussed. Furthermore, no recommendations were made endorsing early VR/Robotic therapy over usual care. Future investigations should test whether rehabilitation using VR/Robotic therapy in the early period post-stroke can influence recovery and to what extent TMS mapping can be used to predict who may benefit most from intervention.

## Ethics Statement

All subjects provided written and verbal informed consents approved by Institutional Review Boards of the New Jersey Institute of Technology, Rutgers University, and St. Joseph's Hospital-Wayne prior to participating.

## Author Contributions

Medical advisement was provided by SM. QQ designed the virtual reality video games used in the interventions. AM, ET, GF, and SA designed the training protocol. MY and ET designed the TMS assessment. Data was collected by JP, MY, QQ, and GF. Data analysis was performed by MY and JP. Manuscript writing was performed by MY and JP equally, and revised by SA, AM, ET, QQ, and GF.

### Conflict of Interest Statement

The authors declare that the research was conducted in the absence of any commercial or financial relationships that could be construed as a potential conflict of interest.
